# Broad and potent neutralization of HIV-1 by human-llama fusion antibodies derived from immunized llamas

**DOI:** 10.1186/1742-4690-9-S2-P94

**Published:** 2012-09-13

**Authors:** LE McCoy, L Rutten, G Dekkers, C Blanchetot, NM Strokappe, A Forsman-Quigley, MS Seaman, H de Haard, T Verrips, RA Weiss

**Affiliations:** 1 University College London, London, UK; 2 Biomolecular Imaging, Department Biology, University of Utrecht, Utrecht, the Netherlands; 3 arGEN-X BVBA, Ghent, Belgium; 4 Biomolecular Imaging, Department Biology, Utrecht University, Utrecht, the Netherlands; 5 Division of Viral Pathogenesis, Beth Israel Deaconess Medical Center, Boston, MA, USA

## Background

Llamas naturally produce heavy chain only antibodies in addition to conventional antibodies. The variable regions of the heavy chain (VHH) demonstrate comparable affinity and specificity for antigens with conventional immunoglobulins. To date, immunizations in human and animal models have yielded only antibodies with limited ability to neutralize human immunodeficiency virus (HIV)-1.

## Methods

A VHH (J3) isolated from a llama multiply-immunized with recombinant trimeric HIV-1 envelope proteins (Env) was found to neutralize 96 of 100 HIV-1 strains, encompassing subtypes A, B, C, D, BC, AE, AG, AC, ACD, CD and G. Isolation involved expression of VHH in E. coli and analysis of neutralization ability in TZM-bl reporter cells.

## Results

Newly isolated VHH from multiple immunized llamas also have broad and potent HIV-1 neutralization activity. J3 targets HIV-1 via the CD4-binding site and neutralization is seen when J3 is used in combination with VHH targeting other Env epitopes. VHH-human FC fusion heavy-chain only antibodies (VHH-FC) have been constructed and J3 activity is not only preserved in this context but enhanced.

## Conclusion

This study shows that experimental immunization with recombinant HIV-1 Env can elicit broad neutralizing heavy-chain only antibodies and supports the development of VHH and VHH-FC as anti-HIV-1 microbicides and therapeutics.

